# Hospital and Institutional News

**Published:** 1914-09-12

**Authors:** 


### HOSPITAL AND INSTITUTIONAL NEWS.

### THE KING'S VISIT TO THE LONDON HOSPITAL.

The King and Queen visited the London Hospital on Thursday, last week, in order to see the wounded soldiers received there, the preparations for whose arrival were described so vividly in the report of Mr. E. W. Morris, the secretary, which we published last week. Arriving between three and four o'clock their Majesties were received by Captain Fenwick, who represented the War Office, Lord Knutsford, chairman, and other hospital officers, and remained about an hour in the institution. They visited every ward in which the wounded were being treated, where every man capable of doing so came to attention, or did his best to perform the military salute. Practically every patient received a word or two from their Majesties, and the details of the more remarkable cases were pointed out. There was great cheering in every ward, and by the time that the King and Queen left several thousand persons had become aware of their presence and gave them hearty greetings when they reappeared. The Union Jack and the Red Cross flag were hoisted on the hospital buildings, but these were the only outward indications that a Royal visit was expected. All who read the account which we published last week will have realised how the sudden influx was prepared for, and how the short notice received by the hospital authorities was successfully coped with, thanks to the devoted work of every section of the staff.

### THE KING AT ST. THOMAS'S HOSPITAL.

As we record in a full account on page 651, some hundred wounded and sick soldiers were received at St. Thomas's Hospital on Thursday, last week. Most of them were medical cases, whose stay in hospital is likely to be relatively short, and the most noticeable feature about them was their exhaustion; "dead tired" was the general description given by a member of the staff. Among them were four from the Royal Army Medical Corps. On Monday, about twelve o'clock, the King and Queen visited the hospital, where they remained about an hour and a half. The three wards occupied by soldiers were visited in turn, the King accompanied by Mr. G. Q. Roberts, the secretary, passing down one side, and the Queen, accompanied by the matron, the other. In this way every patient received a few words. An ophthalmic case on the ground floor was also visited. Their Majesties were informed that two American ladies, Mrs. Garrison and Miss Randell, had sent £5,000 to the funds of the hospital on behalf of the troops. The above, moreover, is not the only inspiring visit which the soldiers at St. Thomas's Hospital have received, for Lord Kitchener also called at the institution last Saturday.

### LORD KITCHENER'S SURPRISE VISITS.

Astonishment and delight were equally manifested last Saturday afternoon in the wards of the London Hospital occupied by the wounded men, when Lord Kitchener suddenly entered. Luckily of the 300 already received little more than half are warded, and Lord Kitchener found 100 or so in the hospital garden enjoying the open air. Beneath Queen Victoria's statue they drew up in double file, and Lord Kitchener congratulated them on the good work which they had already done, and urged them to get well as fast as possible. "We are going to see this thing through," he added, "and want every man." The men answered with three rounds of cheers. On Monday a number of those in the garden on Saturday left for a fortnight's rest in country convalescent homes. For those who are compelled to remain in the wards not only have gifts of tobacco and newspapers been provided, but Mr. E. W. Morris, the tireless secretary, has raised a fund for defraying the expenses of those relatives who wish to come up and see their husbands, sons, and so on in hospital. The necessary money is telegraphed to pay the fares and the cost of a few days' lodgings in London. Lord Kitchener, as we note in our special report on the reception of the wounded at St. Thomas's Hospital, has also visited that institution.

### THE CLASS OF CASE IN NETLEY HOSPITAL.

The Press Bureau issued on Sunday last an interesting statement with regard to the wounded now in Netley Hospital. "At the present time," begins the statement, "Netley Hospital is occupied by some 800 patients, who have been sent home for medical treatment. Some of them are sick, some suffering from sore feet and from various injuries incidental to every campaign, but several have gunshot wounds. It is worthy of note that hardly any of the wounds have been caused by rifle fire, although previous experience would have pointed to rifle wounds as being the commonest. Shrapnel bullets account for nearly all the wounds, and the great majority of those injured are not dangerously wounded. Loss of limbs has so far been very uncommon, if we may generalise from the conditions at Netley." It therefore appears that good recoveries will be made by the great majority of the wounded, who will be able to return to their regiments in a short time. The deduction drawn from the scarcity of rifle-bullet wounds is that the statements to the effect that the German infantry are bad marksmen is correct, and that their aim is uniformly poor. Evidence of the statement that recoveries are likely to be quick is already forthcoming from such centres as Brighton, where convalescent soldiers have been photographed on the beach, and from the convalescent homes in various parts of the country.

### NEW SECRETARY OF THE QUEEN'S HOSPITAL, BIRMINGHAM.

Mr. E. C. Smith, the secretary of the North Ormesby Hospital, Middlesbrough, has been appointed secretary and general superintendent of the Queen's Hospital, Birmingham, in place of Mr. Arthur Hulme, who has resigned, owing to ill-health, as The Hospital recorded in a recent issue. Mr. Smith has been for six years at the Middlesbrough institution, and before that he was for ten years assistant secretary at the Swansea General and Eye Hospital.

### EJECTED HOSPITAL PATIENTS.

Owing to the preparations for the sick and wounded, many institutions and convalescent homes have emptied as many beds as possible, and those late occupants who have been felt capable of it have been returned home. It is unnecessary to add that many of those thus suddenly discharged in an ordinary way would still have been enjoying institutional care, and also that the homes to which they return are frequently unfit to receive those in the early stage of convalescence. Particularly is this the case when, as not infrequently happens, the breadwinner is now out of employment. As a general rule not much voluntary effort seems to have been spent on this unfortunate class, but in one or two centres we are glad to note that Red Cross women workers have arranged a so-called camp hospital, consisting of either suitable cottages or tents, for those in their district thus early discharged "cured." These improvised "hospitals" appear to be staffed by a band of helpers working regularly under a trained nurse, and we refer to the point because such untrained helpers are better employed in looking after the comforts of convalescents than in attempting on a scrap or two of technical knowledge any more ambitious task.

### THE FAMILIES OF ARRESTED GERMANS IN LONDON.

We give elsewhere this week a few points from the annual report of the Metropolitan Asylums Board showing the enormous variety of work, so little realised by the public, which is undertaken by the Managers. A recent instance is perhaps still more remarkable. Last month the Asylums Board received a request from the Local Government Board to undertake the work of providing for the wives and children of Germans who have been arrested in London as alien enemies. The disused but equipped institution of the late St. Giles' Guardians in Endell Street was offered to the Asylums Board for this purpose. As a result the building has been taken over, and a staff has been installed. It is understood that inmates are admitted on police orders. How great the demand on the institution is likely to be cannot be accurately determined, and instead of a standing committee to deal with the work, the chairman and vice-chairman have been empowered to approve whatever further arrangements may be necessary in the matter. There could surely be no better illustration of the multiplicity of work demanded of the Metropolitan Asylums Board, and it is also a remarkable instance of the mutual interdependence of nations even when at war that special provision has to be made for the wives and families of those enemies who happen to be residents and deprived of their means of livelihood through the arrest of the breadwinner of the family.

### CLASSIFYING SANATORIA PATIENTS.

The progress of the tuberculosis work of the Metropolitan Asylums Board has been held up, as everyone knows, by the final adjustment of the schemes which shall comprehend the treatment not only of insured persons, but also of their dependants and of uninsured persons as well. Plans have recently been submitted by the London County Council and duly criticised by the Local Government Board, and it was hoped that a great extension of institutional accommodation and other modes of treatment would be available before the end of the year. How the present crisis will affect these schemes and those being prepared by certain boroughs, it will not be possible to foretell, at least until October next. The sanatoria of the Board at Sutton and at Winchmore Hill still provide the bulk of the accommodation for insured persons. It was, however, decided to proceed with the construction of two sanatoria for men and one for women. Meanwhile, St. George's Home, Milman Street, S.W., has been opened for advanced cases in women. The report of the Downs Sanatorium is full of statistical tables of the usual type, but with this important feature that all the cases are divided according to the classification suggested by Sir R. W. Philip—the now well-known combination of small and capital letters (L, *s*, L, S) which gives twelve (or less if desired) groups into which all cases can be divided with reasonable accuracy. This classification does not appear in the corresponding tables from Winchmore Hill, and thus these are of greatly diminished value and cannot be profitably compared with the other set. Several papers on the subject of tuberculin appear in the medical supplement from the pens of the assistant medical officers. The general impression gained from these papers is that little good has been yet accomplished by tuberculin.

### THE UNRELIABILITY OF CHEAP THERMOMETERS.

Differences of medical opinion as to the value of tuberculin treatment and as to the reliability of the thermometers supplied to the dispensaries were made apparent at a recent meeting of the Derbyshire Insurance Committee. A previous report on the result of visits paid by him to the county dispensaries was emphasised by Dr. J. Court, who declared that the tuberculin treatment was nothing but a huge experiment, and that facts were urgently required on the subsequent history of the patients subjected to it. The medical adviser to the Committee, Dr. S. Barwise, protested that no lay body should be called upon to pass judgment on the value of any methods of treatment. Dr. Court also declared that the clinical thermometers which were supplied to the dispensaries were unreliable. He inquired whether they were of German manufacture, as some of this type were known to jump nearly a degree on a rising temperature. Dr. Barwise disagreed that most of the thermometers were unsatisfactory, and said that it would cost an additional £100 a year to use certified instruments. Almost a year ago, in The Hospital of October 11, 1913 (p. 32), an example was given of the hopeless results which followed at St. Giles' Infirmary, London, on the purchase of cheap thermometers. In this case the Guardians had accepted a contract with a well-known firm of surgical-instrument makers to supply thermometers at 10s. 6d. a dozen. The medical superintendent, Dr. J. B. Cook, at once tested them and found the results recorded by a batch immersed for five minutes in warm water (which was agitated to ensure an even temperature) hopelessly variable. They were returned to the makers, who made an excuse out of the cheap price of the instruments, and sent a further lot, price 18s. a dozen, which they guaranteed to one-fifth of a degree. A similar test applied to these resulted in similar inaccuracies; and, finally, a third lot was also sent in charge of one of the firm's representatives, who was provided with two thermometers tested at Kew. The result once more was a mass of inaccuracies, and the firm's representative retired at last rendered incapable of further expostulation. The moral of this very serviceable warning may well be impressed on the Derbyshire Insurance Committee, which we are glad to see did at least decide to ask the Panel Committee to assist the Sanatorium Sub-Committee in securing a complete record of each case.

### TEMPORARY WARDS AT NEWCASTLE ROYAL INFIRMARY.

The emergency committee in connection with the Royal Victoria Infirmary, Newcastle, which was formed to deal promptly with any difficulties arising out of the war, and of which Dr. G. H. Hume is chairman, has decided to put up immediately two wards, containing fifty beds, which we describe fully on page 655. These wards, it is understood, are temporary, and generous support has been received towards their erection and equipment. News of this decision was published in order to reassure those who feared that, owing to the infirmary's undertaking to reserve absolutely fifty beds for the wounded, and keep 150 filled only with such patients as can vacate them at once if they should be required by the naval and military authorities, the needs of the civil population might suffer. Many of the honorary medical staff are enrolled as officers of the 1st Northern General Hospital, and should their services be required abroad the committee apprehends no difficulty in securing sufficient medical men to carry on the hospital's work.

### HOSPITAL WORK AND SUPPORT IN NEWCASTLE.

It is particularly to be hoped that the working men of Newcastle and the other supporters of the Royal Victoria Infirmary will realise how much additional support is needed by the institution at the present time. Newcastle may be singled out especially in this respect since it is reported to be more free from distress than any other great industrial centre. The reason is that Newcastle industries are primarily those of engineering and shipbuilding, and the well-known Elswick works of Armstrong, Whitworth's, for instance, alone employ 20,000 men. These and the other firms of the city are now working day and night on Government orders, and will probably remain so for as long as the war lasts. On the other hand, the building industry has been hit, and the Northumberland coal trade is feeling severely the loss of its export trade to Germany. Such unemployment as exists in the city itself, however—and the average is actually lower than it was in 1912 and very slightly higher than last year—has also been mitigated by the number of recruits, 10,000, who have joined Lord Kitchener's armies. Thus Newcastle probably holds not only the record in recruiting of any city in proportion to its population, but also the record in prosperity during war-time. The bearing of these facts on the Royal Infirmary's position is obvious: while the institution is making exceptional efforts and, as we note, has formed an emergency committee specially to deal with them, the support of the working classes, especially in view of their prosperity, should be proportionately generous. This is an exceptional case, and for this reason is worth particular attention.

### THE KING AND HIS WOUNDED.

To the visit paid by the King and Queen to the London Hospital must now be added the fact that for several days in succession the King has devoted his afternoons to visiting the wounded officers and men at Sister Agnes' Hospital for Officers, and at the Herbert Hospital at Woolwich, whither he went on Saturday afternoon. The Royal Herbert Hospital is a military hospital, containing 636 beds, and the King, who was wearing the undress uniform of a Field-Marshal, was shown over the wards by Colonel Simpson. The King stayed an hour in the institution, in which some three hundred men of the Expeditionary Force are under treatment, and sympathetic inquiries were made by both the King and Queen. It will add to the cheering effect which their visits have already produced—being indeed the King's proof of his farewell message to his troops in which he said that their welfare would never be far from his thoughts—that the Royal visits are not likely to be limited to the London district. It is understood that, if circumstances permit, the King and Queen will also visit some of the provincial centres at which the wounded are being received. We can well believe the enthusiasm which would be excited, say, at Cambridge by such a personal act of the Sovereign.

### SUGGESTED NEW NAME FOR THE METROPOLITAN ASYLUMS BOARD.

In the recently issued report of its work for the year 1913, the Metropolitan Asylums Board draws attention to the fact that its name very inadequately indicates the manifold duties entrusted to it. These duties have greatly increased since 1867, when the Board was constituted to provide accommodation for the relief of paupers suffering from fever, smallpox, or insanity of a chronic and harmless character. Nowadays in addition, the Board provides for non-pauper cases; for cases of diphtheria, measles, whooping-cough, and puerperal fever; for insured persons suffering from tuberculosis; for non-certified mental defectives; for convalescent and sick children, including those suffering from ophthalmia and ringworm; it possesses a training-ship for the training of boys for sea service, and it is the central authority for dealing with the casual poor of London. No wonder the Board is dissatisfied with its name, and there is much to be said for its suggestion that a better descriptive title would be that of The Metropolitan Board of Public Assistance.

### THE REOPENING OF HERTFORD COUNTY HOSPITAL.

By the time that this issue is in the hands of our readers the Hertford County Hospital will have reopened for the reception of patients. It will be recalled that in the middle of July it was decided to close the wards and out-patient department of the institution in order that certain sanitary improvements might be carried out. A new drainage system, comprising iron pipes four inches in diameter, has been designed under the superintendence of Messrs. Rogers, Field and Bean, of London, and has been carried out at a cost of about £600 by Messrs. Fitch and Cox, of Enfield. The new system is practically complete and the patients return to-day (Saturday). At the time that it was decided to undertake this sanitary improvement scheme the subscribers were warned not to give in-patient letters except in cases of really urgent necessity. Even in these instances an inquiry had to be made in order that a bed might be available in the temporary quarters which the hospital committee had arranged. Thanks to the co-operation and assistance of the Master and Council of Haileybury College, which is, of course, quite close to Hertford, a portion of the school sanatorium has been placed at the disposal of the hospital authorities for the reception of urgent cases. Here the accommodation was inevitably limited, but it was readily taken advantage of for the benefit of urgent cases, and it is from the sanatorium that the patients returned on Friday last. The Secretary, Mr. Albert Stoddart, has been kept very busy during the installation of the new drainage system, and, no doubt, he and the committee, to say nothing of the patients and the public generally, will be very glad to get back to work in their old quarters now that the improvement has been carried out.

### DUBLIN HOSPITALS AND THE WAR.

At the Royal City of Dublin Hospital a special meeting of governors was held last week to decide what answer should be given to a request from the military authorities to name the number of beds which it could place at the disposal of the sick and wounded. It was decided to set aside thirty—the number in the institution is 124. In this connection it may be added that the hospital has already felt the effects of the war, through having lost a number of its staff. In fact, the house surgeon, the assistant physician and pathologist, the medical officer in charge of the *x*-ray department, and his colleague in charge of the throat and nose department have all left. Also one of the members of the visiting staff is now at the front with the first field hospital. These facts are interesting, since the suggestion has been put forward that the Dublin hospitals are not able to accommodate many of the sick and wounded, and one of the resulting proposals has been to convert the buildings used for the recent civic exhibition to hospital purposes. We do not know how far it may apply to Dublin, but Belfast, in view of its recent active hospital preparations in connection with the Ulster Volunteers, ought to be in a particularly forward position with hospital preparations. This indeed should be more or less the case with the principal towns.

### DEVELOPMENTS AT THE LONDON HOSPITAL.

In view of the recent developments at the London Hospital the tin building in the garden has been used as the temporary quarters for the resident physicians and surgeons during certain building alterations in the hospital. The alterations to the cardiac department have been postponed for the present, but the alterations to the laundry are proceeding, however, and also the erection of the salvarsan wards. In connection with the heart department, a promise is announced from Mrs. Paterson of a sum of about £20,000 to be invested "for the investigation and problems connected with the heart's working and relief." Moreover, the lease of a large plot of land at the back of Tredegar House has been purchased for the sum of £400, and this piece of land makes a valuable addition to Tredegar House in affording opportunities for recreation of the London Hospital pupil-probationers. There will be sufficient room for two or three tennis courts. In view of the anxiety concerning supplies in consequence of the war, it was decided to make a rule not to admit patients who were suffering from ailments which were not urgent, and by this means beds became gradually vacant, and fell from about 820 to 600 beds in daily occupation. During this anxious period Mr. E. W. Morris, the secretary, was absent on holiday, and the responsibility devolved upon the assistant secretary, Mr. Elliott. We are glad to see that at the recent quarterly Court acknowledgment was made of his services. There was at this time some fear with regard to the delivery of goods, apart from the contractors' readiness to supply them. This was due to the commandeering of horses and motor lorries. The Boy Scouts, however, promptly offered their services, and were ready to haul goods to the hospital in hand-carts from all parts of London.

### A HOSPITAL CHAPLAIN AND THE CHOLERA EPIDEMIC.

At the quarterly court of the London Hospital reference was made to the death of one of the vice-presidents of the hospital—Canon Thomas Scott, of Felixstowe. Canon Scott was chaplain of this hospital in 1866 and went through the great cholera epidemic about that time. Since his retirement at Felixstowe he has been of very great help to the hospital in taking services for the patients at the convalescent home at Felixstowe on Sunday evenings. These services are keenly appreciated by the patients.

### DISTINCTION FOR A MEDICAL SUPERINTENDENT.

The King has been pleased to sanction the promotion of Dr. J. A. Sutherland from Honorary Associate to Knight of Grace in the Order of the Hospital of St. John of Jerusalem. This promotion may be regarded as a recognition of Dr. Sutherland's services in connection with the Order, with which he has been associated for some twenty-five years. In fact, he has conducted classes and been a lecturer and examiner for many years. Dr. Sutherland, who is a graduate of Edinburgh University and medical superintendent of the infectious hospital under the North Bierley Joint Hospital Board, has received its congratulations. He was elected an Honorary Associate last April, and has largely helped to secure the efficiency of the movement in his district. Among others receiving the same distinction is Surgeon-General Sir John May, R.N.

### INFIRMARY OFFICER ON ACTIVE SERVICE.

Dr. George T. Cregan, M.B., Ch.B., of St. Pancras South Infirmary, London, has volunteered for active service with the Red Cross. Dr. Cregan, who was formerly house surgeon at Stanley Hospital, Liverpool, graduated M.B., Ch.B. at Victoria University, Manchester, in 1910.

### THE AMALGAMATION OF MANCHESTER UNIONS.

An interesting side-light upon the effect on building schemes of amalgamation between different Boards of Guardians comes from Manchester. Three Unions are on the eve of amalgamation, and one of them, comprising the South Manchester Guardians, has decided to build a children's hospital. The scheme, of course, is a relatively expensive one. The decision of the South Manchester Guardians to carry it out was discussed last week by the Manchester Board of Guardians, who expressed disapprobation. Mr. Russell remarked that while he did not wish to interfere with the doings of a neighbouring authority, yet one of the principal arguments in favour of amalgamation was that it would represent a reduction in expenditure. To involve the ratepayers in an expenditure of some £50,000 was not fair. He therefore moved that the Local Government Board be asked not to consent to any further expenditure by the South Manchester Guardians for providing a children's hospital beyond what was necessary for their own immediate requirements. The resolution was supported by several speakers on the ground that the proposed expenditure was wasteful. We can hardly believe that the South Manchester Guardians will be pleased at the above discussion, and, in the absence of evidence to the contrary, we imagine that whether or not South Manchester needs a publicly supported children's hospital is a question which is not likely to be much affected by administrative schemes of amalgamation. The need or absence of need is an outstanding fact, and, pending the establishment of one body for the city, the local authorities on the spot ought to be the best judge of its necessity.

### NAVAL SICK LANDED AT ABERDEEN.

The arrangements for the reception of sick and wounded at Aberdeen were tested by the arrival on August 29 of the hospital ship *Rohilla*. Forty-four men arrived sick, not wounded, and comprised twenty-four surgical cases and twenty medical cases. The *Rohilla*, which is stated to be a vessel of 7,365 tons, was met by two tugs, and the men, except those able to walk, were stretched on canvas slings wrapped in blankets. Within a quarter of an hour those thus incapacitated were transferred to ambulance vans or motor-cars improvised for this purpose. Their kits followed in Co-operative motor waggons. Colonel Scott-Riddell M.V.O., district commissioner of the Red Cross Society, the Lord Provost, Dr. Williamson, Major McLennan, R.A.M.C., Captain Smart, R.A.M.C., and other medical men were early on the quay. The men were then promptly removed to the Royal Infirmary, where they were received by the Railway Servants' Ambulance Corps, in the charge of Mr. R. Duguid, joint station superintendent. There were also six squads of stretcher-bearers at the infirmary from the local section of the St. Andrew's Ambulance Corps; and the whole process of transference to the institution was accomplished in half an hour. Hardly also had the *Rohilla* sick been disembarked when the *Granite City* was sighted with Prince Albert on board. He was also lying in a sling, and when carried on shore was met by Sir James Reid, Fleet-Surgeon Toman, the Lord Provost, and a trained nurse, in whose charge he was taken in a police-ambulance van to the Northern Nursing Home.

### PROGRESS OF THE WORKERS' FUND AT CAMBRIDGE.

The Cambridge and District Workers' Hospital Fund recently held its fourth annual meeting in the Out-patient Waiting-hall at Addenbrooke's Hospital. Mr. W. J. Dyball, chairman of the society, presided, and the President, Colonel T. W. Harding, referred to the strange times through which we are all passing, when most of the honorary physicians and surgeons appear in khaki and the matron, sisters, and nurses appear in their military nursing uniforms. The work of the past two years, both in connection with the Workers' Fund and the hospital, had been carried on under difficulties. There were signs that these difficulties will increase, but the Workers' Fund members must go forward. The general committee of the hospital were deeply grateful to every member of the Workers' Hospital Fund for the handsome contribution annually for the past four years and for the increased interest. Their help was never more necessary than at the present time. The number of centres at the close of the present year was 97, as compared with 91 at the close of last year. During the year sixteen new centres had been established, including one each at Christ's College, Downing College, and Magdalene College. The contributions for the past year from the various centres plus personal subscriptions amounted to £596, and after the deduction of expenses there was a net balance of £586 for allocation. Of this sum Addenbrooke's Hospital receives seven-tenths and an additional grant of £50, making a total of £460. Mr. W. J. Dyball, the chairman, referred to the valuable assistance which the committee had been able to render to a large number of the workers and members of their families by sending them to convalescent homes or sanatoria, and by supplying requisite appliances, and he emphasised the value of the convalescent fund which enabled the committee to help members in the way mentioned. We are glad to record this progress, as we have chronicled the society's work since its inception, and have urged the possibility of workers' funds in centres like Cambridge, where the shop takes the place of the factory, which is the home of the workers' funds in the industrial towns.

### HOSPITAL WILLS.

Nothing is more remarkable than the way in which the voluntary system has attracted support to itself from the public and has almost succeeded in making the voluntary hospitals, as it were, the residuary legatee of all testators who are anxious to bequeath their wealth to public purposes and have often no very individual preference of their own. It is not too much to say that the first claim which flashes across every such man's mind is that of the hospitals. Thus the late Mr. George Ivey, whose estate has been sworn at £63,000 odd, apart from bequests of £500 each to the City of London Lying-in Hospital and to St. Bartholomew's Hospital, has left residue of his property in equal shares to about thirty charitable institutions. It is true that many charities besides voluntary hospitals have a place in this list, but we believe, nevertheless, the fact to be true that it is indirectly through the work and affection with which the hospitals are regarded in this country that all forms of charity have benefited. The voluntary system of hospital support has established on wide lines and almost on a religious basis the principle of voluntary help for others less fortunate than ourselves, and without the example which it has set we believe that the general stream of charity would be much less generous and constant than it is to-day.

### NATIONAL MEDICAL UNION AND THE WAR.

The unity of the medical profession in its desire to afford every assistance to the nation during the war is well indicated by a resolution which was passed at a meeting of the National Medical Union last Saturday. The resolution was to the effect that the Council of the Union was prepared to submit a list of its members who are willing to place their services in various capacities in their districts at the disposal of the War Office. It is of interest to note that this list contains men who are anxious to do any work, whether of an interesting or uninteresting character; and consequently shows on the part of the Union a recognition of the fact that to remain at home may, for those debarred from active service, be as patriotic and useful as to go abroad. There is much necessary work to be done, and we trust that the authorities will make use of the National Medical Union's offer.

### PANEL CHEMISTS AND THE COST OF DRUGS.

Panel chemists who are under contract with Insurance Committees to dispense medicines and supply drugs at prices which even at normal times do not yield more than a very small profit have been placed in a somewhat unfortunate position by the general advance in the market quotations for the majority of the drugs on the tariff. It is satisfactory to know, however, that the Insurance Commissioners have appreciated the difficulties, and after considering the question in all its aspects have agreed to make special arrangements in connection with the prices of certain drugs included in a special schedule. Among the drugs included in the original schedule are potassium bromide, ammonium bromide, sodium bromide, bismuth carbonate and other salts of bismuth, acetyl-salicylic acid, salicylic acid, and sodium salicylate. Additions will probably be made to the list from time to time, but the Commissioners make it quite clear that any concession it may be found possible to make would not in any case apply to drugs which, for whatever causes, have not advanced beyond the range of ordinary market fluctuations, or to drugs which have advanced in price for causes independent of the present war conditions. Any question of additional payments must be confined to the case of certain drugs shown to the satisfaction of the Government to have advanced in price considerably by reason of the war.

### THIS WEEK'S DRUG MARKET.

Further advances in the prices of synthetic drugs have taken place, and the rising tendency in other products of Continental origin is maintained. Phenacetin is again much dearer; acetyl-salicylic acid is now five times the normal price; Russian cantharides are almost unobtainable and buyers would be willing to pay famine prices; cream of tartar shows a further substantial advance. Business is naturally very quiet, and all transactions are of a very restricted character. It is satisfactory to know that various products which have hitherto been obtained from Germany are already being manufactured in this country, and it may be expected that in course of time the situation will be relieved considerably. In the meantime the small supplies of a number of commonly used drugs are steadily diminishing, and prescribers are no doubt turning their attention to suitable substitutes. Reference to standard works on therapeutics should suggest methods by which economy might be effected without entailing any harmful results. No public sales of drugs have been held in Mincing Lane for some time, and it is not probable that the usual auctions will be resumed at present.

